# Antiinflammatory Activity of Aqueous Extract of *Stereospermum kunthianum* (Cham, Sandrine Petit) Stem Bark in Rats

**DOI:** 10.4103/0250-474X.51943

**Published:** 2009

**Authors:** F. P. Ching, E. K. I. Omogbai, S. O. Okpo, R. I. Ozolua

**Affiliations:** Department of Pharmacology, Faculty of Basic Medical Sciences, College of Health Sciences, Niger Delta University, WilberforceIsland, Yenagoa, Bayelsa State, Nigeria; 1 Department of Pharmacology and Toxicology, Faculty of Pharmacy, University of Benin, Benin City, Edo State, Nigeria

**Keywords:** *Stereospermum kunthianum*, antiinflammatory activity, paw oedema, leucocytes, exudate, carrageenan, rats

## Abstract

*Stereospermum kunthianum,* Cham, Sandrine Petit (family: Bignoniaceae) is used in traditional medicine to treat bronchitis, pneumonia and coughs, gastritis, wounds, rheumatic arthritis, ulcers, dysentery, leprosy and venereal diseases in humans. The antiinflammatory activity of the aqueous extract of the stem bark was investigated with experimental animal models using the carrageenan-induced paw oedema, leucocytes migration and granuloma air pouch tests in rats. The extract (100, 200 or 400 mg/kg) at 3 h post-treatment caused a significant (p<0.05) reduction in the paw oedema in rats. The effect of the extract was most pronounced at the dose of 400 mg/kg and was higher than that of indomethacin (10 mg/kg). The extract (400 mg/kg) caused a significant (p<0.05) reduction in the number of recruited leucocytes and it's inhibition of peritoneal exudate formation was comparable to that of indomethacin at a dose of 10 mg/kg. The exudate formation inhibited by 400 mg/kg of the extract in the granuloma air pouch test was comparatively less to that of indomethacin at a dose of 10 mg/kg. The findings of the study indicate that the aqueous extract of *Stereospermum kunthianum* stem bark possesses antiinflammatory activity which is probably related to the inhibition of prostaglandin synthesis. This is a possible rationale for its folkloric use as an antiinflammatory agent.

Most people in the rural areas of the world depend largely on herbs for the treatment of several ailments because medicinal herbs constitute indispensable components of traditional medicine practice due to low cost, easy access and ancestral experience[1]. Inflammatory responses are associated with most pathological disorders and many Nigerian traditional medicine practitioners enjoy huge patronage and success in this area[2]. Although a good number of plant species are used for this purpose, scientific and pharmacological information on them is scarce or very little[3]. *Stereospermum kunthianum* has a reputed history of its use in inflammatory situation[4,5]. It is known as *sansami* among the Hausa of North Nigeria, *umana* among the Tiv of Middle Belt of Nigeria, ayada among the Yoruba of South West Nigeria and *alakiriti* among the Igbo of South East Nigeria. The efficacy of the water extract of *Stereospermum kunthianum* in human complement system fixation *in vitro* has been reported[6]. Antiplasmodial activity of naphthoquinones and one anthraquinone from the lipophilic extract of the root bark of *Stereospermum kunthianum* has also been reported[7]. No previous scientific information was found on its antiinflammatory activity to support it's used in traditional medicine in inflammatory situation. This study investigated its antiinflammatory activity with experimental animal models using the carrageenan-induced paw oedema, leucocytes migration and granuloma air pouch tests in rats.

The fresh stem bark of the *Stereospermum kunthianum* was collected in Idi-Okpe, Ogun State, Nigeria in March, 2006. Identification and botanical authentication were done by Mr. Usang Felix Inah (plant taxonomist) of the Forestry Research Institute of Nigeria, Ibadan where a voucher specimen (No. FHI 107277) was deposited for future reference.

The stem bark was carefully separated from the woody part, cut into small pieces, sun-dried and pulverized using a grinder (Laboratory Mill, serial NO. 4745, Christy and Norris Ltd, England). The powdered material (400 g) was macerated in 2 l of distilled water at an initial temperature of 60°, allowed to cool and filtered after 24 h. The filtrate was evaporated to dryness in an oven set at 40° until a constant weight was obtained. The yield was 26.4% with reference to the powdered stem bark. The extract obtained was stored in closed containers in the refrigerator at −4° till required.

Carrageenan and indomethacin were obtained from Sigma Chemical Co. (St. Louis, MO, USA). Disodium hydrogenorthophosphate and potassium dihydrogenorthophosphate (BDH Chemicals Ltd, Poole, England) were used. Heparin injection (Glaxosmithkline, England) was purchased from the University of Benin Teaching Hospital (UBTH), Benin City, Nigeria. Egg albumin (Merck, Germany) was obtained from the Department of Biochemistry, University of Benin, Nigeria.

Approval for the use of animals for antiinflammatory experiments had been obtained from the ethical committee of the Faculty of Pharmacy, University of Benin, Nigeria. Wistar rats of either sex obtained from the Animal House unit of the Department of Pharmacology and Toxicology, Faculty of Pharmacy, University of Benin, Benin, Edo State, Nigeria were used. The animals maintained under standard laboratory conditions (12 h light and dark cycles) had free access to standard chow (Bendel Flour Mills and Feeds, Plc. Ewu, Nigeria) and water.

Acute toxicity was performed according to the OECD-423 guide lines[8]. Oral acute toxicity was studied in overnight-fasted rats with water *ad libitum.* Adult Wistar rats of either sex (110-160 g) were randomly allocated into groups of five animals per group. The rats were administered orally distilled water (5 ml/kg), control or aqueous extract (1, 2, 3, 4, 5, 6, 7 or 8 g/kg.) of *Stereospermum kunthianum* stem bark. Besides the number of deaths, other parameters such as agility, muscular tonus, tremors, convulsions, feed and water intake, breathing patterns and presence of mouth secretions were observed for the first 12 h and for further 14 days. If mortality was not observed, the procedure was repeated for a higher dose till a maximum dose of 8 g/kg was attained.

Carrageenan-induced paw oedema method used was as previously described[9]. Rats were randomly allotted to groups of five animals per group. The animals were administered orally, distilled water (5 ml/kg), extract (100, 200, or 400 mg/kg) or indomethacin (10 mg/kg). One hour later, 0.1 ml of 1% w/v carrageenan in normal saline was injected into the plantar aponeurosis of the right hind paw of the rat. Prior to the injection of the carrageenan, the right hind paw of each rat was measured by means of vernier calipers. Measurements were repeated at hourly interval for a maximum of six hours after carrageenan injection. The difference between the initial paw size and the paw size at each post treatment time point was used to estimate the degree of oedema.

Carrageenan-induced leucocytes migration method was performed as previously described[10] was used. Rats (fasted overnight) were randomly allotted to groups of five animals per group. They were administered by oral route, distilled water (5 ml/kg), extract (400 mg/kg) or indomethacin (10 mg/kg) one hour before intraperitoneal injection of 0.1 ml of carrageenan, 1%w/v in normal saline. Three hours later the rats were sacrificed by excess chloroform inhalation. An incision was made on the peritoneal cavity of each rat and 5 ml of phosphate buffered saline (containing 1.38 g/l of disodium hydrogenorthophosphate; 0.19 g/l of potassium dihydrogenorthophosphate; 8.0 g/l of sodium chloride; 5.0 iu/ml of heparin and 3% egg albumin) was injected. After gentle massage of the peritoneum, the exudate was aspirated with a sterile syringe. The exudate volume (minus the wash fluid, 5 ml) as well as the total leucocytes count was determined[10].

The granuloma air pouch test of Selye[11] was used. Wistar rats of either sex (145-160 g) were randomly allocated into groups of five animals each. The dorsal skin surface of the rats was shaved and disinfected with 70% ethanol. Twenty millitres of air was injected subcutaneously under ether anaesthesia at approximately the midpoint of the animals' back using a hypodermic syringe with the needle directed towards the scapular region. The animals were administered distilled water (5 ml/kg, p.o.), extract (400 mg/kg, p.o.), or indomethacin (10 mg/kg, s.c.). One hour later for the extract and thirty minutes for indomethacin treated animals respectively, 0.5 ml of carrageenan 10% w/v in olive oil (sterilized by heating) was injected into the air pouch of each rat. Remnant air was removed from the air pouch 24 h after the injection of the carrageenan. The rats were treated daily with the same dose of extract or indomethacin for five days with the last dose on the fifth day given one hour prior to the time the animals were sacrificed. The animals were sacrificed by inhalation of excess chloroform. The air pouch was carefully opened and the volume of exudate formed in each animal was measured. Data are expressed as mean±SEM. or in simple percentage and analyzed using the Student's t-test. Results were considered significant when p<0.05.

The observation of the animals after oral acute treatment with aqueous extract of *Stereospermum kunthianum* stem bark (1, 2, 3, 4, 5, 6, 7 or 8 g/kg) did not reveal any signs of toxicity and no deaths were observed even at the highest dose of 8 mg/kg body weight. The effect of the extract on carrageenan-induced paw oedema is presented in Table 1. In the animals administered only distilled water, the subplantar injection of carrageenan produced a local oedema that increased progressively from 6.35±0.40 mm after the first hour to reach a maximum (8.05±0.55 mm) within 4 h. The extract (100, 200, or 400 mg/kg) as from the third hour post-carrageenan injection caused a dose dependent and significant (P<0.05) reduction in oedema in the rats compared with the same time of the distilled water treated group. Indomethacin (10 mg/kg) produced a significant (P<0.05) decrease in oedema at the fourth hour compared with the same time of the distilled water treated group.

**TABLE 1:** EFFECT OF AQUEOUS EXTRACT OF *STEREOSPERMUM KUNTHIANUM* STEM BARK ON CARRAGEENANINDUCED PAW OEDEMA

Treatment	Paw Oedema (mm)						
	
	0 h	1 h	2 h	3 h	4 h	5 h	6 h
Distilled water (5 ml/kg)	4.95±0.05	6.35±0.40	7.00±0.18	7.15±0.12	8.05±0.55	7.90±0.49	7.50±0.29
*S. kunthianum* (100 mg/kg)	4.25±0.25	5.50±0.20	6.13±0.31^a^	6.25±0.25^a^	6.43±0.22^a^	6.25±0.25^a^	6.25±0.25^a^
*S. kunthianum* (200 mg/kg)	4.63±0.24	5.88±0.52	6.50±0.29	6.38±0.24^a^	6.25±0.25^a^	6.25±0.25^a^	6.25±0.25^a^
*S. kunthianum* (400 mg/kg)	5.00±0.41	5.50±0.35	6.28±0.28	6.25±0.25^a^	6.25±0.25^a^	6.2±0.20^a^	6.25±0.25^a^
Indomethacin (10 mg/kg)	4.88±0.31	6.30±0.24	6.55±0.32	6.40±0.37	6.25±0.25^a^	6.25±0.25^a^	6.13±0.31^a^

Values are Mean±SEM.

a P < 0.05, significantly different from that of the same time of the distilled water group; Student's t-test (n=5 per group)

The aqueous extract was studied for its ability to inhibit leucocytes migration to the peritoneal cavity in rats (Table 2). Intraperitoneal injection of carrageenan induced formation of 0.9 ±0.18 ml of peritoneal exudate containing 3.26±0.09×10^9^ leucocytes/l in the distilled water treated rats. Treatment with extract (400 mg/kg) or indomethacin (10 mg/kg) prior to the injection of the carrageenan reduced the peritoneal exudate volume by 24.73% and 26.88% of control (0.70±0.10 ml and 0.68±0.15 ml, respectively). The number of recruited leucocytes in the extract or indomethacin treated rats were significantly (p<0.05) lower compared to that of the distilled water treated rats. The number of recruited leucocytes was reduced by 27.61% and 52.76% (2.36±0.32 and 1.54±0.04×10^9^ leucocytes/l of control, respectively).

**TABLE 2:** EFFECT OF AQUEOUS EXTRACT OF *STEREOSPERMUM KUNTHIANUM* STEM BARK ON CARRAGEENANINDUCED LEUKOCYTES MIGRATION IN RATS

Treatment	Exudate vol. (ml)	Inhibition (%)	WBC×10^9^/l	Inhibition (%)
Distilled water (5 ml/kg)	0.93±0.18	-	3.26±0.09	-
*S. kunthianum* (400 mg/kg)	0.70±0.10	24.73	2.36±0.32^a^	27.61
Indomethacin (10 mg/kg)	0.68±0.15	26.88	1.54±0.04^a^	52.76

Values are mean±SEM of five experiments.

a P <0.05, significantly different from the distilled water group, Student's t-test.

The results of the effect of the extract in the air pouch test in rats are presented in Table 3. Five days after injection of carrageenan (in olive oil), the volume of the exudate produced in the air pouch of the distilled water group were 1.44±0.21 ml. Daily treatments with the extract (400 mg/kg/d) reduce the exudate volume by 39.58% (0.87±0.14 ml) of the distilled water group. Indomethacin (10 mg/kg/d) given subcutaneously reduced the exudate volume by 68.06% (0.4±0.03 ml) of the distilled water treated rats.

**TABLE 3:** EFFECT OF AQUEOUS EXTRACT OF *STEREOSPERMUM KUNTHIANUM* STEM BARK ON CARRAGEENAN-INDUCED EXUDATE FORMATION IN RATS

Treatment	Exudate volume (ml)	Inhibition (%)
Distilled water (5 ml/kg)	1.44±0.21	-
*S. kunthianum* (400 mg/kg)	0.87±0.14	39.58
Indomethacin (10mg/kg)	0.46±0.03^a^	68.06

Values are mean±SEM, of 5 determinations

a p <0.05, significantly different from the distilled water group, Student's t-test

Inflammation is a complex and dynamic condition in which many changes take place at the site of inflammation as well as systemically. It involves a complex array of enzymes activation, release of mediators, extravasation of fluid, migration of cells, tissue breakdown and repair[12]. It is known that the acute inflammatory response consists of three main vascular effects viz. vasodilatation and increased vascular flow, increased vascular permeability and leucocytes migration to the injured tissues[13]. It is also known that antiinflammatory effects can be elicited by a variety of chemical agents and there is no remarkable correlation between their pharmacological activity and chemical structure[14]. This coupled with the complexity of the inflammatory process, makes the use of several different experimental models necessary when conducting pharmacological trials.

The present study establishes the antiinflammatory activity of the aqueous extract of *Stereospermum kunthianum* stem bark in a number of experimental rat models, which represent different phases of inflammation. The extract produced a dose-dependent antiinflammatory effect on carrageenan-induced paw oedema. The extract (400 mg/kg) produced a marked antiinflammatory effect at the third hour post-carrageenan injection, which was higher than that produced by indomethacin (10 mg/kg). Carrageenan-induced oedema is a model of acute inflammation used in the study of non-steroidal antiinflammatory drugs[15]. The model is suitable for evaluating the antioedematous effect of natural products and is believed to be biphasic. The first phase which occurs within an hour is believed to involve the release of serotonin and histamine while the second phase which occurs after one hour has been attributed to prostaglandin and the continuity between the two phases is provided by kinin[15]. That the extract produced marked antiinflammatory effect 3h post-carrageenan injection suggests that its antiinflammatory activity may involve the inhibition of prostaglandin synthesis and cyclooxygenase products since the carrageenan inflammatory model basically reflects the action of prostaglandins[16]. The extract prevented formation of exudate and leucocytes mobilization induced by intraperitoneal injection of carrageenan. The carrageenan-induced leucocytes migration assay has been adjudged as an excellent acute and sub-acute model for the measurement of fluid extravasation, leucocytes migration and other biochemical parameters which accompany inflammatory stimuli[17]. Production of exudate in this model is related to local release of histamine, kinins and synthesis of prostaglandins[17]. Migration of leucocytes would not be directly related to cyclooxygenase products, but the process is inhibited by non-steroidal antiinflammatory compounds indicating that many mechanisms may be implicated in its control[18]. The inhibitory effect of the extract on the intraperitoneal formation of exudate and leucocytes mobilization is probably due to the inhibition of prostaglandins. This possibility is reinforced by the fact that the extract at the same dose (400 mg/kg) remarkably inhibited paw oedematous process which is believed to be mediated by prostaglandins.

The extract at a dose of 400 mg/kg inhibited exudate formation in the granuloma air pouch test which was comparatively less than that of indomethacin after five days of pretreatment. The air pouch exudate formation is an exudative, sub-acute type of inflammation used to estimate the potency of antiinflammatory corticosteroids both after local and systemic application. Although other short-term inflammatory mediators beside prostaglandins may be involved in the granulomatous model, however; drugs that reduce the synthesis of prostaglandins also decrease the exudate formation[19]. The results from the air pouch model may favour the possible inhibition of prostaglandin synthesis as one of the possible mechanisms of antiinflammatory activity of the extract. On the basis of this study, it is concluded that the aqueous extract of *Stereospermum kunthianum* stem bark possesses antiinflammatory activity, which is probably related to the inhibition of prostaglandin synthesis. Also the study scientifically justifies the folkloric use of the plant in inflammatory situation. Further studies are in progress to elucidate the possible mechanism(s) of action of the antiinflammatory activity of the aqueous extract of *Stereospermum kunthianum* stem bark.
